# Conference report on the 28th annual meeting of the European Musculo-Skeletal Oncology Society, 29 April–1 May 2015, Athens

**DOI:** 10.3332/ecancer.2015.550

**Published:** 2015-07-08

**Authors:** Andreas Leithner, Dimosthenis Andreou, Robert Grimer, Stefano Ferrari, Georg Gosheger, Panayiotis J Papagelopoulos, Stefan S Bielack

**Affiliations:** 1 Department of Orthopaedic Surgery, Medical University of Graz, Graz 8036, Austria; 2 Department of Orthopaedics and Tumour Orthopaedics, University Hospital Muenster, Muenster 48149, Germany; 3 Oncology Department, Royal Orthopaedic Hospital, Birmingham B31 2AP, UK; 4 Chemotherapy Department, Istituto Ortopedico Rizzoli, Bologna 40136, Italy; 5 1st Department of Orthopaedics, University of Athens, Medical School, University General Hospital ATTIKON, Athens 124 62, Greece; 6 Klinikum Stuttgart, Olgahospital Paediatrics 5 (Oncology, Hematology Immunology), Stuttgart 70174, Germany

**Keywords:** conference report, sarcoma, multidisciplinary treatment, surgery, chemotherapy

## Abstract

The 28th Annual Meeting of the European Musculo-Skeletal Oncology Society was organised in Athens by the local host Professor Papagelopoulos and his team. The main objective of the meeting was to focus on recent advances in the diagnosis and treatment of bone and soft tissue sarcomas. The interdisciplinary nature of the meeting was of great value—surgeons, oncologists, pathologists, radiologists, and basic researchers discussed new strategies in the war on sarcoma. This report will highlight the major findings of this successful meeting.

The ‘European Musculo-Skeletal Oncology Society’ (E.M.S.O.S.) aims at advancing the science and practice of the diagnosis and multidisciplinary treatment of bone and soft tissue tumours, to promote basic and clinical research, and to disseminate knowledge in order to provide a common high standard of musculo-skeletal oncology. The particular purpose of the Society is to promote mutual collaboration between different specialists and institutes involved in the treatment of musculo-skeletal tumours. The society was founded in 1987 and has held annual meetings ever since.

The 2015 conference took place in Athens, the cradle of the Western world, a city nowadays troubled by a serious financial crisis. Despite these economic and social problems, the local hosts impressed with a highly interesting programme and perfect organisation leading to the largest meeting so far with 510 participants from 45 countries. The 181 oral presentations and 207 poster presentations were chosen from 398 abstracts from 37 countries. The conference was supported by six international societies (e.g. EFORT, ESMO, ESTRO, and CTOS) and three national societies.

With the welcoming words from the local organiser Professor Panayiotis Papagelopoulos and his brief review of the last 50 years of progress in bone and soft tissue sarcoma treatment, the conference started with the traditional training day including most of the 37 invited lectures on state-of-the-art diagnostic and treatment modalities. Basic principles in imaging techniques, pathology and treatment strategies as well as recent discoveries were presented to the interdisciplinary audience. One of the highlights of the training day was John Healey’s (Memorial Sloan Kettering Cancer Centre, New York, U.S.A.) lecture on new developments in orthopaedic oncology, focusing on targeted treatment options, and immune checkpoint therapy. In this context, Professor Healey also placed emphasis on the parallel progression model of primary cancers and metastases, a theory that a primary cancer begins to seed metastatic cells in other organs in its early growth stages, which go on to acquire distinct sets of mutations [16]. This theory might help explain why the sequencing of the primary tumour does not always help identify effective targeted treatments for metastatic disease. Another highlight was the lecture of Professor Rob Grimer (ROH, Birmingham, UK), the Society’s former President, who focused on the need for early detection of soft tissue sarcomas [11] and presented on the on-going Sarcoma Awareness Campaign which is taking place in the United Kingdom.

In the evening, the ‘Hippocrates lecture’ was delivered by Meletios A Dimopoulos, Rector of the National and Kapodistrian University of Athens, themed on his field of expertise—multiple myeloma [6]. The opening ceremony that followed was attended by the Greek Minister of Health and Social Solidarity, Panagiotis Kouroumblis. In his fiery speech, he called for the delinking of drug pricing from the pharmaceutical companies’ research and development costs and outlined that primary healthcare, public health, and the chronically ill will be the priorities of the country’s health system in the following years. Athanassios S Fokas, Chair of Nonlinear Mathematical Science, University of Cambridge and Academy of Athens, and a world renowned mathematician [7] then fascinated and puzzled the audience with his thoughts on perception and the importance of unconscious processes.

## Pelvic and sacral tumours

The second day started with three parallel sessions, one on local treatment options and outcome of spinal and pelvic tumours, one included the nurses’ programme with further state-of-the art presentations, and one session focused on systemic treatments. Among several other interesting topics, M Bus from Leiden presented the results of an international multicentre study on the LUMiC prosthesis for reconstruction after resection of periacetabular tumours [3]. The study demonstrated that while dual-mobility cups helped reduce the rate of dislocation, infection remains a major problem in pelvic surgery.

Surgery is not always the best local treatment option, as T Kunisada and coworkers (Department of Orthopaedic Surgery, Okayama University Hospital, Japan) described in their presentation on sacral chordoma treatment using carbon ion radiotherapy. They concluded that this emerging technique should be used for the local treatment of chordomas localised in or above the third sacral vertebra [18]. This message was underlined by a subsequent presentation on the preliminary results of a prospective trial regarding efficacy of carbon ion therapy for sacrum chordoma and mobile spine chordoma from a group from Italy (S Bandiera, V Vitolo and coworkers, Rizzoli Orthopaedics Institute, Bologna and Centro Nazionale Adroterapia Oncologica, Pavia, Italy), demonstrating a good clinical, radiographical, and histological tumour response to treatment.

F Gouin and coworkers (University of Nantes, Nantes, France) highlighted the high risk of a postoperative deep infection following the resection of periacetabular bone sarcomas. In a study of 43 patients, the authors demonstrated that the risk of deep infection in the first 6 months following surgical treatment amounted to 30%. The development of deep infection led to a significant increase of the length of hospital stay, with 45 days compared to 23 days for patients without infections, and a worse functional outcome. About 38% of the infected patients had a chronic infection at last follow-up. A higher number of blood transfused units and older age were associated with the development of infection in this study, while the length of the operation and the size of the tumour were not.

The risks associated with high blood loss during pelvic and sacral tumour surgery were the focus of another study by A Freeman and coworkers (Royal Orthopaedic Hospital, Birmingham, United Kingdom), who evaluated the influence of hypotensive epidural anaesthesia (HEA) on the risk of bleeding. The authors were not able to demonstrate a significant benefit for patients undergoing HEA compared to patients undergoing standard anaesthesia, with both groups having similar intraoperative mean arterial pressures, mean intraoperative blood loss and mean blood units transfused in the first 24 hours after the operation.

## New results from chemotherapy trials

In the Oncology session, Stefano Ferrari, (Rizzoli Orthopaedics Institute, Bologna, Italy) the current president of EMSOS, focused on the toxicity profile associated with the EURO.B.O.S.S. trial—a European international protocol for bone-sarcoma patients older than 40 years [5]. The analysis of 307 evaluable patients showed that the median received chemotherapy dose in this age group was lower than the planned dose and that only 28% of the patients were able to complete the treatment without dose reduction. There was a strong relation between age and chemotherapy compliance, and a higher incidence of febrile neutropenia and transfusion support was observed in female patients. He concluded that the EURO.B.O.S.S. protocol is feasible, but a high risk of renal toxicity has been observed (41%) in this age group and that overall the incidence of renal and peripheral neurotoxicity is higher compared to the one observed in younger patients.

A further highlight was Francoise Rédini’s (INSERM UMR 957, Nantes, France) talk on how to overcome TRAIL resistance in paediatric bone sarcoma models [9, 17]. Osteosarcoma and Ewing’s sarcoma cell lines often show resistance to the pro-apoptotic cytokine TNF-related apoptosis inducing ligand (TRAIL) that can electively kill tumour cells. To overcome this resistance, several strategies including trimeric TRAIL presentation at the surface of carrier mesenchymal stem cells stably transfected with full-length human TRAIL and a novel TRAIL-receptor agonist (APG880) were presented.

Later on, Alessandra Longhi (Rizzoli Orthopaedics Institute, Bologna, Italy) presented the preliminary results of an EMSOS wide study on extraskeletal osteosarcoma. The overall survival (OS) rate of 147 analysed patients, so far, was 55%. Patients with localised disease had an OS of 62% compared to 28% for those with metastatic disease. Chemotherapy appeared to be more beneficial for patients with a tumour size >5 cm, but no definitive results regarding the value of systemic treatment could be drawn so far. Other centres were encouraged to join this study.

The traditional EMSOS lecture was given by Stefano Ferrari, EMSOS’ current president. He pointed to various developments in the field of targeted therapies for some of the rarest subtypes of soft tissue sarcomas, which in the past were treated only surgically. One of these tumours is alveolar soft part sarcoma (ASPS), which accounts for 0.2–0.9% of all soft tissue sarcomas and most commonly affects patients between the ages of 15 and 35 years. ASPS has a high rate of both early and late metastases and is considered to be resistant to standard chemotherapy protocols. This tumour is characterised by the presence of a specific chromosomal translocation encoding the chimeric transcription factor (ASPL-TFE3) that activates expression of MET [19]. As a result, crizotinib, a multitargeted tyrosine kinase inhibitor is currently being studied in patients with locally advanced and/or metastatic ASPS in a phase II trial of the European Organisation for Research and Treatment of Cancer (EORTC). Furthermore, following gene expression profiling studies conducted on surgical samples of ASPS, which revealed upregulation of several transcripts associated with angiogenesis, cell proliferation and metastasis, cediranib, an orally bioavailable, small-molecule inhibitor of all three vascular endothelial growth factor receptor (VEGFR-1, VEGFR-2, and VEGFR-3) tyrosine kinases was shown to be active in active in patients with metastatic ASPS and is currently being compared to sunitinib, another multitargeted tyrosine kinase inhibitor, in a phase II trial of the National Cancer Institute (NCI) [14]. Tyrosine kinase inhibitors are also being evaluated in other rare sarcomas considered to be resistant to standard chemotherapy regimens, like clear cell sarcoma and extraskeletal myxoid chondrosarcoma, offering new treatment options in patients with advanced disease. In his conclusion, Professor Ferrari underlined the importance of an interdisciplinary cooperation that is mandatory in the field of sarcoma treatment.

In the afternoon, one had to choose between parallel sessions on surgical topics concerning the upper extremity, focusing on scapula, clavicle and extra-articular shoulder joint resections, and minimally invasive techniques, such as preoperative embolisation, core needle biopsies versus fine needle aspiration, vertebroblasty, and alcohol injection for aneurysmal bone cysts. These sessions were followed by one of the highlights of this conference—a joint plenary session of several European Interdisciplinary Sarcoma Groups. As we have not seen major advances in the treatment of osteosarcomas and Ewing sarcomas in the last 30 to 40 years, the session focused on the future goals of clinical research. Representatives of the Soft tissue and Bone Sarcoma Committee of the EORTC, the Scandinavian Sarcoma Group (SSG), the French Sarcoma Group (FSG), the Italian Sarcoma Group (ISG), the Spanish Sarcoma Group (GEIS) the Cooperative Osteosarcoma Study Group (COSS), and the EuroEwing Consortium presented their ideas and thoughts on how to fight sarcoma. Representing COSS Stefan Bielack (Klinikum Stuttgart, Olgahospital Paediatrics 5 (Oncology, Hematology Immunology), Stuttgart, Germany) again addressed the controversy on the usage of Mifamurtide [13] and regretted that there was no drug being made available for an large-scale international prospective randomised trial with this agent.

In the next session, Georg Gosheger (Department of General Orthopaedics and Tumour Orthopaedics, University Hospital Muenster, Muenster, Germany) presented the concept of a ‘mobile arthrodesis’, an invention of his which is designed to allow different fixed knee positions in patients following arthrodesis of the knee and is currently being developed for market release in the near future. Busy evening sessions on metastatic bone disease and skeletal reconstruction topics were concluded by two lectures—one by Duncan Whitwell (Oxford University Hospitals NHS Trust, Oxford, United Kingdom) on the best way to reconstruct massive bone defects and another by Sofia Thoma, United Kingdom on the usage of expandable prostheses in children. Following the EMSOS General Assembly, the evening ended with a lovely meeting dinner in the beautiful Aegli Zappeiou.

## Allograft reconstruction for bone defects

Nonetheless, the last day of the meeting started early with parallel sessions on allograft reconstruction, soft tissue tumours and the nurses’ programme. The subject of reconstruction of massive bone defects using allografts and/or autografts was dominated by two of the most important men in this field, Rudolfo Capanna (Ortopedia Oncologica e Ricostruttiva, Azienda Ospedaliera Universitaria Careggi, Florence, Italy) [4] and D Luis Muscolo (Hospital Italiano de Buenos Aires, Buenos Aires, Argentina) [1]. Both are regarded as pioneers of biological reconstruction methods, with Professor Capanna being the inventor of the ‘Capanna-technique’ using allografts in combination with a vascularised fibula for the reconstruction of massive bone defects. In the Campanacci Lecture D Luis Muscolo highlighted the latest developments in the field of three-dimensional preoperative planning and resection in orthopaedic oncology [15] and called for mentorship in order to preserve medical knowledge and enthusiasm. Muscolo emphasised the need for thorough preoperative virtual surgical planning, particularly concerning the selection of osteotomy sites and he underlined that the outcome of massive allografts reconstruction could be improved with adequate anatomic matching, infection prevention, modern internal fixation, and stable soft tissue reconstructions.

This talk was followed by the Enneking Lecture, which was presented this year by one of the grand masters of orthopaedic oncology, Franklin Sim from the Mayo Clinic, Rochester, MN, USA. In his talk Professor Sim presented a detailed overview of recent advances in the management of soft tissue sarcomas. The next lecturer, Wei Guo, Musculoskeletal Tumour Centre, People’s Hospital, Peking University, Beijing, China impressed with high numbers of pelvic resections and his renowned expertise [10].

## Denosumab for giant cell tumour of bone

Later, in the afternoon sessions, on topics like cartilaginous tumours, paediatrics and quality of life led to lively discussions. Furthermore, several interesting studies on the treatment of giant cell tumour of bone were presented. F Traub (Division of Orthopaedic Surgery, Department of Surgery, Mount Sinai Hospital, Toronto, Canada) presented the results of a prospective study regarding the efficacy of Denosumab with special reference to its role in joint preservation. Twenty patients with locally advanced giant cell tumours of bone underwent treatment with denosumab for at least 6 months prior to surgical management. All of the tumours demonstrated some positive response to denosumab. All six patients with fractures through the subchondral bone prior to treatment showed fracture healing under treatment. Reappearance of the subchondral bone following treatment with denosumab strongly favoured the prospects of joint preservation. About 17/20 patients underwent joint salvage treatment with thorough curettage, intralesional high-speed burring, and allograft or cement reconstruction. The presence of residual giant cells on histopathology did not appear to be associated with the risk of local recurrence—which occurred in three patients. The authors concluded that denosumab appears to be a useful adjuvant in the treatment of patients with giant cell tumour of bone especially to facilitate joint preservation and reduce explicitly the extent of surgery.

Another study by G Beltrami and coworkers (Ortopedia Oncologica e Ricostruttiva, Azienda Ospedaliera Universitaria Careggi, Florence, Italy) evaluated the results of a series of 18 patients with giant cell tumour of bone surgically managed between 2010 and 2014, which were treated with denosumab for median time of 5.7 months before and 6 months after surgery. The preservation rate of native joint function in this study was 94.4%, while two of the 18 patients relapsed and restarted denosumab treatment after a median follow-up of 12.3 months. The authors concluded that denosumab is effective in downstaging of tumour size, leading to less invasive surgical procedures and a good preservation rate of joint function. W Ebeid (Cairo University, Cairo, Egypt) reported on 17 patients with giant cell tumour of bone treated with denosumab, six of which had pulmonary implants at presentation. A significant regression of these implants was demonstrated in three patients.

Regarding possible risk factors for local recurrence after surgical treatment of giant cell tumour of bone, N Lujic and coworkers (Institute for Orthopaedic Surgery ‘Banjica’, Belgrade, Serbia) analysed tissue samples from 164 giant cell tumours. The authors demonstrated that p53 expression in mononuclear cells was the most significant predictive factor for local recurrence with a hazard ratio of 6.2 (*p* < 0.001). The expression of Cyclin D1 in giant cells, containing less than 15 nuclei, was also significantly associated with the risk of local recurrence with a hazard ratio of 8.4 (*p* = 0.038). If these results can be verified in a separate patient cohort, the might resolve the dilemmas in the therapeutic approach to giant cell tumour of bone.

Another highlight of the meeting was Michelle Ghert’s (Division of Orthopaedic Surgery, Department of Surgery, McMaster University, Hamilton, Ontario, Canada) lecture on the Prophylactic Antibiotic Regimens in Tumour Surgery (PARITY) trial, the first ever international, multicentre, prospective randomised study in the field of orthopaedic oncology [8]. The study aims at comparing the rates of deep infection in endoprosthetic reconstruction after resection of lower-limb tumours between two prophylactic antibiotic durations, 24 hours and 5 days postoperatively, after a previous survey of orthopaedic oncologists showed that the practice of antibiotic prophylaxis varies greatly among different centres [12]. Approximately, 100 patients have already been enrolled in 18 centres worldwide, while another 27 centres are ready to begin with patient enrolment. A total of 600 patients will be enrolled until the end of the study.

In one of the last presentations of the meeting, Jay Wunder (Division of Orthopaedic Surgery, Department of Surgery, Mount Sinai Hospital, Toronto, Canada) gave an interesting talk on CD146 as a newly identified cell surface marker of tumour propagating cells in human osteosarcoma and undifferentiated pleomorphic sarcoma cells. Serial transplantation assays of CD146+ cells from these tumours in immunocompromised mice demonstrated that this cell population is highly tumorigenic and can sustain tumour growth over multiple passages. The transcriptional profiling of these cells revealed a common activation of several signalling pathways including TGF-beta and Notch. Inhibition of the Notch pathway using a γ-secretase inhibitor significantly reduced tumour growth and self-renewal in both tumour types, suggesting possible novel therapeutic options to reduce tumour recurrence for patients with these high-grade sarcomas.

## Satellite Symposia

The conference accommodated three satellite symposia presented by Amgen, Glaxo Smith Kline, and Implantcast presenting novelties in the fields of RANK ligand inhibition, clinical trials in soft tissue sarcoma, and megaprostheses, respectively.

## Prizes

A Longhi from the Istituto Ortopedico Rizzoli, Bologna, Italy won the prize for the best paper presentation with a multicentre study on extraskeletal osteosarcoma. She presented the preliminary results of this retrospective EMSOS study and invited further centres to include their patients.

F Redini (Nantes Atlantique University, Nantes, France) was awarded with a prize for the best basic science presentation—based on a work on TRAIL-based therapeutics in osteosarcoma: involvement of bone tumour microenvironment in TRAIL resistance [9].

The prize for the best poster presentation went to M Susa and coworkers from Keio University, Tokyo, Japan for their work on CT-guided cryoablation for locally recurrent or metastatic bone and soft tissue tumour.

WP Bekkering from the Leiden University Medical Centre, Leiden, the Netherlands won the prize for the best nurse paper presentation with a long-term follow-up study on the quality of life after malignant bone cancer surgery—in relation to her previous publication [2].

## Next meeting

The EMSOS 2016 meeting will be held in La Baule, Nantes, France from 25–27th May—with a special focus on Ewing’s sarcoma, margins in sarcoma, pelvic bone tumours, and targeted therapy.

## Conclusion

Bone and soft tissue sarcoma treatment demands an interdisciplinary approach that was mirrored and intensified by the EMSOS meeting in Athens. Several targeted agents are currently evaluated in patients with rare soft tissue sarcoma subtypes, previously thought to be resistant to systemic therapy. The rapid development of new treatment options in these patients highlights the need for the discussion of cases in interdisciplinary tumour boards. The fact, however, that we have not seen major advances in the prognosis of patients with osteosarcoma and Ewing’s sarcoma for nearly 40 years demands novel clinical trials, either in improving current treatments or in testing promising novel strategies.
